# Diagnostic efficacy of [^99m^Tc]Tc-PSMA SPECT/CT for prostate cancer: a meta-analysis

**DOI:** 10.1186/s12885-024-12734-4

**Published:** 2024-08-08

**Authors:** Qi Wang, Svea Ketteler, Shamim Bagheri, Ali Ebrahimifard, Markus Luster, Damiano Librizzi, Behrooz H. Yousefi

**Affiliations:** 1 Department of Nuclear Medicine, University Hospital Marburg, Philipps University Marburg, Baldinger-Strasse, 35043 Marburg, Germany; 2 Tongji Shanxi Hospital, Shanxi Bethune Hospital, Shanxi Academy of Medical Sciences, Third Hospital of Shanxi Medical University, Taiyuan, 030032 China; 3 Center for Tumor Biology and Immunology (ZTI), Core Facility Molecular Imaging, Philipps University Marburg, Hans-Meerwein- Str. 3, 35043 Marburg, Germany

**Keywords:** Prostate cancer, Technetium, PSMA, SPECT/CT, Meta-analysis

## Abstract

**Background:**

Prompt and accurate diagnosis of prostate cancer (PCa) is of paramount importance for effective treatment planning. While Gallium-68 labeled prostate-specific membrane antigen (PSMA) positron emission tomography (PET)/computed tomography (CT) has proven efficacy in detecting PCa, limited availability poses challenges. As a potential alternative, [^99m^Tc]Tc-PSMA single photon emission computed tomography (SPECT)/computed tomography (CT) holds promise. This systematic review and meta-analysis aimed to evaluate the diagnostic value of [^99m^Tc]Tc-PSMA SPECT/CT for prostate cancer.

**Methods:**

A comprehensive search of PubMed, Cochrane, EMBASE, Scopus, Ovid, and Web of Science databases was conducted until July 2024. Sensitivity and specificity data were extracted to assess the diagnostic accuracy of [^99m^Tc]Tc-PSMA SPECT/CT, while the Quality Assessment of Diagnostic Accuracy Studies (QUADAS-2) tool was used to evaluate study quality. Statistical analyses were performed using STATA 18, with MetaDisc 1.4 employed to detect threshold effects. Diagnostic accuracy indicators, including sensitivity, specificity, diagnostic odds ratio (DOR), negative likelihood ratio (LR-), and positive likelihood ratio (LR+), were pooled. The area under the curve (AUC) of the combined model was calculated using summary receiver-operating characteristic (SROC) curves.

**Results:**

Seven studies meeting the inclusion criteria were identified from an initial pool of 1467 articles, with no publication bias observed. The pooled sensitivity, specificity, and AUC of [^99m^Tc]Tc-PSMA SPECT/CT were found to be 0.89 (95% CI, 0.84–0.93), 0.92 (95% CI, 0.67–0.99), and 0.93 (95% CI, 0.90–0.95), respectively. Additionally, the comprehensive diagnostic odds ratio, diagnostic score, positive likelihood ratio, and negative likelihood ratio were calculated as 95.24 (95% CI, 17.30-524.41), 4.56 (95% CI, 2.85–6.26), 11.35 (95% CI, 2.31–55.71), and 0.12 (95% CI, 0.08–0.18), respectively.

**Conclusions:**

In conclusion, our findings demonstrate that [^99m^Tc]Tc-PSMA SPECT/CT exhibits favorable diagnostic performance for prostate cancer and can provide valuable supplementary information, particularly in regions and settings where [^68^Ga]Ga-PSMA PET/CT availability is limited, such as remote areas. These results highlight the potential of [^99m^Tc]Tc-PSMA SPECT/CT as a valuable tool in the diagnosis and management of prostate cancer, warranting further investigation and validation in larger patient cohorts.

**Supplementary Information:**

The online version contains supplementary material available at 10.1186/s12885-024-12734-4.

## Background

Prostate cancer (PCa) is the second most common malignancy and the fifth most common cause of cancer-related deaths in men worldwide, accounting for over 29% of all cancers diagnosed in 2023 and causing approximately 350,000 deaths [1–4]. Early detection, improved cancer screening, and ensuring access to cancer treatment are important factors to improve prostate cancer prevention. According to a recent report from the US National Cancer Statistics, early detection and treatment of prostate cancer reduced prostate cancer mortality, with age-adjusted prostate cancer mortality rates declining each year [4, 5].

Prostate-specific membrane antigen (PSMA) is a type II transmembrane glycoprotein, whose structure consists of a large extracellular structural domain, a trans-membrane protein, and an intracellular structural domain, and whose extracellular portion accounts for 95% of the protein, making it an ideal target for small molecule and antibody drugs used in imaging and therapeutics [6, 7]. PSMA is overexpressed in prostate tumor cells, and the presence of PSMA is 100- to 1000-fold higher in prostate cancer than in benign prostate tissue or normal tissue of most other organs. PSMA expression increases with cancer stage and grade [8–10], especially in metastatic prostate cancer, making PSMA the most important biomarker for diagnosis and targeted therapy [10, 11].

With the development of molecular imaging, positron emission tomography (PET) has achieved great success in the development of tracers and biomarkers for a wide range of biological targets and has an undisputed superiority in molecular imaging. [^68^Ga]Ga-PSMA-HBED-CC (called PSMA-11) is the most widely used and prominent ^68^Ga-labelled PSMA radioligand in clinical practice worldwide and has a high diagnostic accuracy [12]. Although the higher sensitivity and spatial resolution of PET technology compared to single photon emission computed tomography (SPECT) technology has made PET more popular than SPECT, the higher cost of PET equipment makes it sometimes difficult to use it in routine clinical practice, especially in remote areas [13]. Gallium-68 can be obtained by elution of a Germanium-68/Gallium-68 (^68^Ge/^68^Ga) generator. These generators can be locally eluted, facilitating the production of radiolabeled PSMA; however, the high cost of ^68^Ge/^68^Ga generators and the fact that the amount of radionuclide obtained per elution is only sufficient for a relatively small number of patients. The short half-life of ^68^Ga may impact on the large-scale introduction of [^68^Ga]Ga-PSMA PET/CT into clinical practice [14]. With significant progress in SPECT hardware and software, SPECT is gradually closing the gap with PET technology. Meanwhile, compared to ^68^Ga production, ^99^Mo/^99m^Tc generators are not only capable of producing enough ^99m^Tc to support large-scale clinical use, but are also relatively inexpensive, making ^99m^Tc an ideal choice for radiopharmaceutical applications [15]. At the same time, ^99m^Tc has optimal emission characteristics for single photon emission computed tomography imaging [16]. The simplicity and low cost of SPECT using ^99m^Tc radiopharmaceuticals with a long half-life has led to the worldwide adoption of this technique as part of routine diagnostic procedures in nuclear medicine [17]. Therefore, [^99m^Tc]Tc-PSMA SPECT/CT may have broader clinical applicability than [^68^Ga]Ga-PSMA PET/CT, which may provide uro-oncologists and nuclear medicine physicians with more options in the diagnosis of prostate cancer, especially when [^68^Ga]Ga-PSMA PET/CT is not yet widely available.

Previous studies have reported the diagnostic efficacy of [^99m^Tc]Tc-PSMA SPECT/CT for prostate cancer. [18–20] The systematic combination of diagnostic accuracy indicators of the included studies in a meta-analysis leads to a more reliable conclusion. Thus, the purpose of this study is to assess the clinical value of [^99m^Tc]Tc-PSMA SPECT/CT for the diagnosis of prostate cancer by systematic review and meta-analysis of published studies. The project was registered at INPLASY (https://inplasy.com) (registration number INPLAS 202420065).

## Methods

### Literature search strategy

Bibliographic databases PubMed, Cochrane, EMBASE, Scopus, Ovid, and Web of Science were searched until July 2024. The search included combinations of the following terms: (1) Prostate Neoplasms; (2) Technetium; (3) Tomography, Emission-computerized, Single-photon; (4) Sensitivity or Specificity or Diagnosis or Staging. The search keywords were as follows: (1) “Prostatic Neoplasms” OR “Prostate Cancer” OR “Prostate Carcinoma”; (2) “Tomography, Emission-computed, Single-photon”; (3) “Technetium” OR “Technetium-99m” OR “Tc-99m”; (4) “Sensitivity” OR “Specificity” OR “Diagnosis” OR “Neoplasm Staging”.

### Inclusion and exclusion criteria

Retrieved publications were subject to the inclusion and exclusion criteria established below.

Inclusion criteria: (1) [^99m^Tc]Tc-PSMA SPECT/CT was used to diagnose prostate cancer patients; (2) Studies assessed the diagnostic sensitivity and specificity can use histopathology or/and imaging or clinical follow-up as a reference standard; (3) Patient-based research; (4) Sufficient data were presented to calculate the true-positive (TP), false-positive (FP), false-negative (FN), and true-negative (TN) values for the imaging techniques.

Exclusion criteria: (1) Review or meta-analysis, case report; (2) The data provided by the article was not enough to calculate the diagnostic accuracy; (3) The research content was irrelevant to this study; (4) Publications with study population overlap; (5) Patients with a secondary malignant neoplasm were excluded from the study.

### Documentation and evaluation

The following data were extracted from the included studies by two independent researchers: author, publication time (year), country, study design (prospective or retrospective), sample size, TP, FP, FN, TN, sensitivity, specificity, accuracy, tracer type, and the diagnostic gold standard, prostate cancer stage at the time of patients’ enrollment. Disagreements between the two reviewers were resolved by discussion or consultation with a third reviewer. The Quality Assessment of Diagnostic Accuracy Studies (QUADAS-2) scale [21] including four areas: case selection, index test, reference standard, and flow and timing has been used to assess the methodological quality and risk of bias of the selected studies. The low, high, or unclear risk was assessed for each domain.

### Statistical methods

STATA 18 was used for statistical calculations in this study. Meta Disc 1.4 was used to detect threshold effects. Diagnostic accuracy indicators, including sensitivity, specificity, diagnostic odds ratio (DOR), negative likelihood ratio (LR-), and positive likelihood ratio (LP+), were pooled. The area under the curve (AUC) of the combined model was calculated using summary receiver operating characteristic (SROC) curves. The heterogeneity among the included studies was quantified using Galbraith radial plot. We assessed the publication bias of the included studies using Deek’s funnel plot. All hypothesis tests were statistically significant with a two-sided P-value of less than 0.05.

## Results

### Study characteristics

**Fig. 1:**
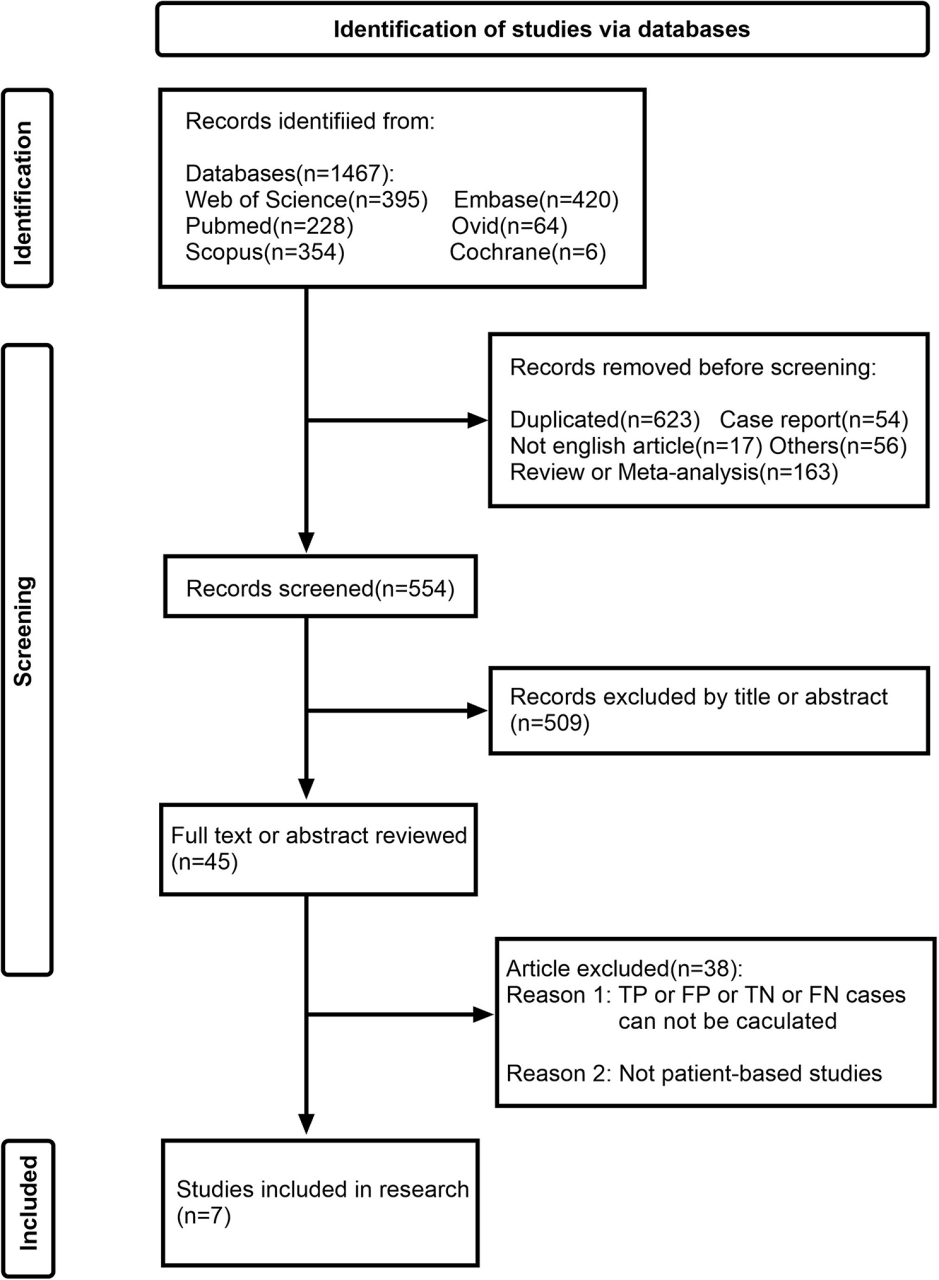
PRISMA flow chart of the study selection process

A total of 1467 relevant publications were generated through the systematic search. Following screening based on the inclusion/exclusion criteria, this meta-analysis included a total of 7 publications comprising 4 conference abstracts [22–25] and 3 full texts [26–28]. Figure 1 displays the PRISMA flowchart about the inclusion of studies. The sample size ranged from 30 cases to 152 cases. Two studies were conducted in China, one in India, one in Bulgaria, two in Hungary, and one in the US and Europe. A total of six radiotracers were included in the seven studies; these were [^99m^Tc]Tc-MIP-1404, [^99m^Tc]Tc-PSMA-I&S, [^99m^Tc]Tc-PSMA-T4, [^99m^Tc]Tc-PSMA-11, [^99m^Tc]Tc-mas3-ynal-k(Sub-KuE), and [^99m^Tc]Tc-HYNIC-PSMA. Two studies had a retrospective study design, four had a prospective study design, and one did not state the study design. Five studies used histopathology as the final diagnostic criterion, one study used histopathology or follow-up as the final diagnostic criteria, and one study did not state the reference standard. Six articles had patients in the primary stage and one article had patients in the recurrent stage. The characteristics of the included studies are shown in Table 1.

**Table 1:** Characteristics of the 7 included studies

Studies	Year	Country	Sample size	TP	FP	FN	TN	Sensitivity	Specificity	Accuracy	Study design	Tracer	Final diagnostic criteria	Stage
Slawin KM	2014	US & EU	54	49	0	3	2	94%	100%	94.4%	Prospective	[^**99m**^**Tc]Tc-MIP-1404**	Histopathology	Primary
Farkas I	2020	Hungary	30	20	0	5	5	80%	100%	83.3%	Retrospective	[^**99 m**^]**Tc-mas3-ynal-k(Sub-KuE)**	Unclear	Primary
Sergieva S	2021	Bulgaria	36	27	0	5	4	84%	100%	86.1%	Prospective	**[** ^**99m**^ **Tc]Tc-PSMA-T4**	Histopathology/Follow-up	Recurrent
Agrawal K	2022	India	30	16	1	2	11	88.9%	91.6%	90%	Prospective	**[** ^**99m**^ **Tc]Tc-PSMA-11**	Histopathology	Primary
Gao Y	2022	China	152	86	16	11	39	88.7%	70.9%	82.2%	Unclear	**[** ^**99m**^ **Tc]Tc-HYNIC-PSMA**	Histopathology	Primary
Zhang Y	2023	China	56	43	3	1	9	97.7%	75.0%	92.9%	Prospective	**[** ^**99m**^ **Tc]Tc-HYNIC-PSMA**	Histopathology	Primary
Farkas I	2024	Hungary	48	24	0	4	20	86%	100%	92%	Retrospective	**[** ^**99m**^ **Tc]Tc-PSMA-I&S**	Histopathology	Primary

**Fig. 2:**
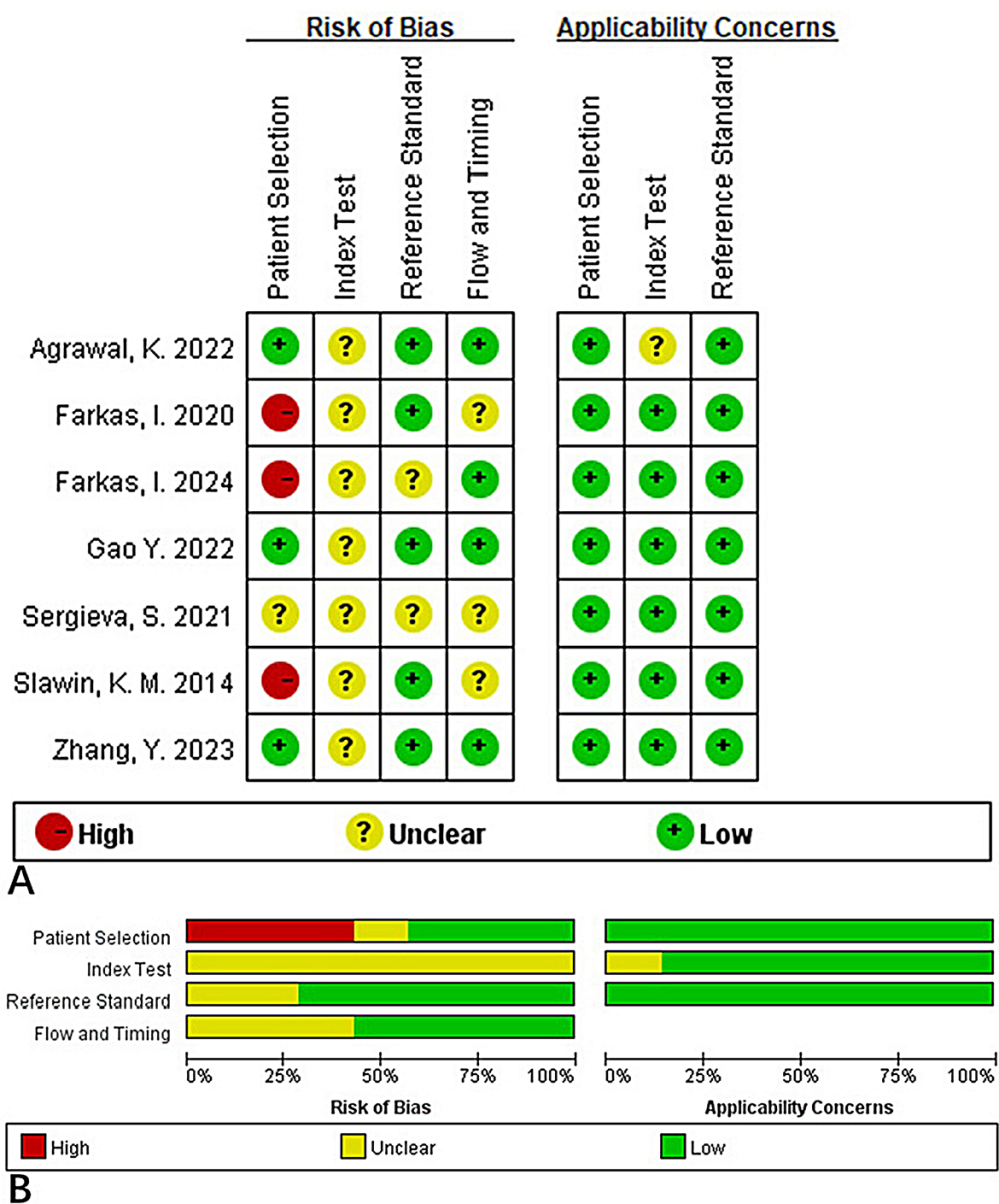
Sectional bar chart summarizing the quality assessment of the literature using a diagnostic tool for assessing the qualities of experimental research: (**A**) Methodological quality of the included studies (individual assessment); (**B**) Summary of the methodological quality of the included studies

The RevMan 5.4 was used to plot a segmented bar chart containing the scoring criteria for each QUADAS-2. Figure 2 shows that the quality of the included literature was high. The majority of the literature included a description of the gold standard used and a description of the diagnostic criteria for prostate cancer.

### Meta-analysis

#### Heterogeneity analysis

**Fig. 3:**
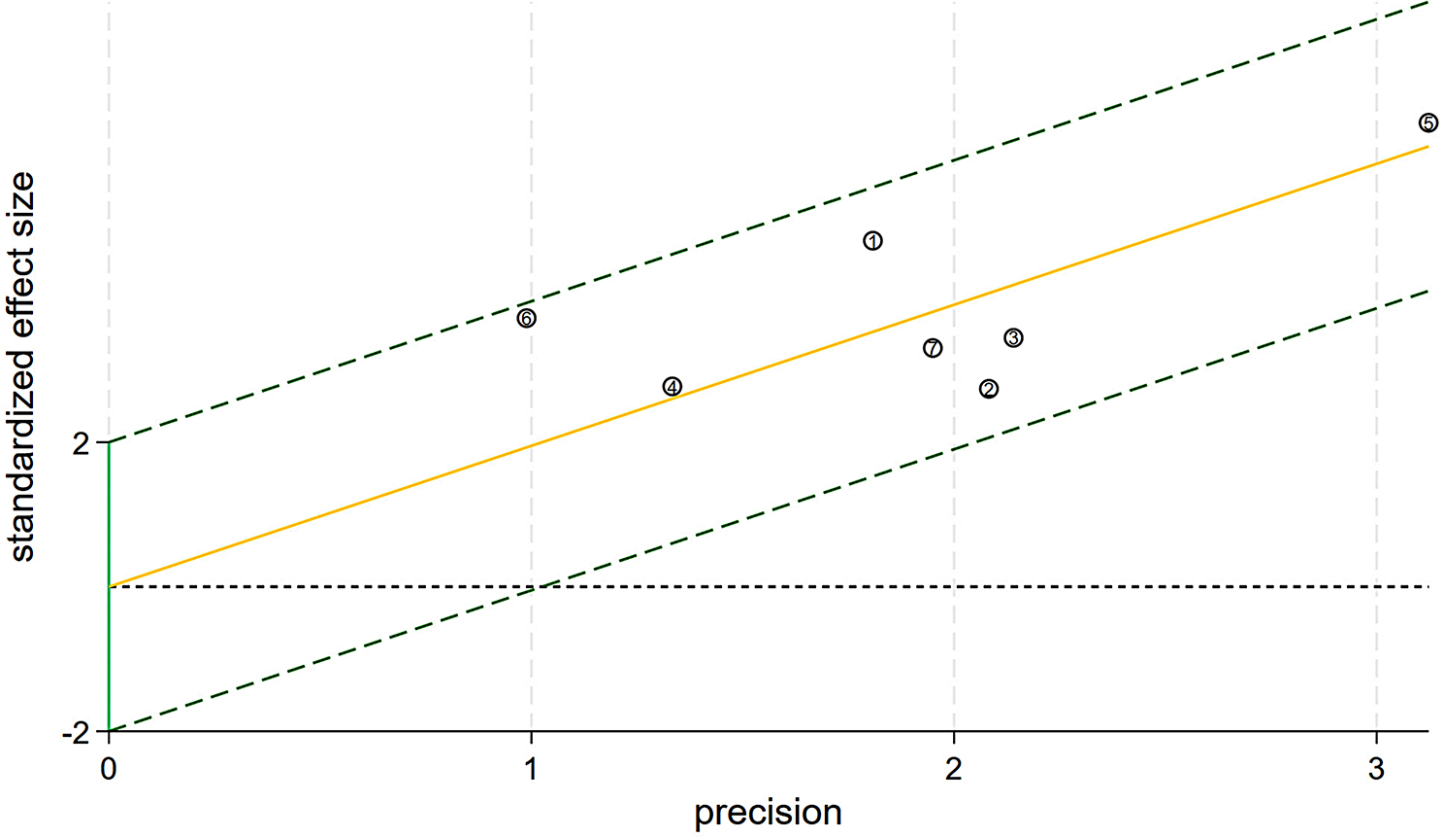
Galbraith radial plot: all studies fell within the confidence interval regression line, proving that the data could be merged

There was no no threshold effect (Spearman correlation coefficient was 0.505, *p* = 0.248). The Galbraith radial plot showed the data could be merged (Fig. 3).

#### Combined effect analysis

**Fig. 4:**
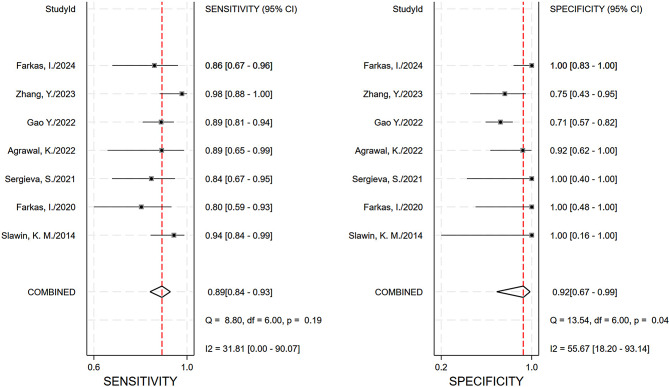
Combined sensitivity and specificity forest plot. There is no obvious heterogeneity in either of them, for sensitivity: *I*^*2*^ = 31.81, *p* = 0.19, for specificity: *I*^*2*^ = 55.67, *p* = 0.04

**Fig. 5:**
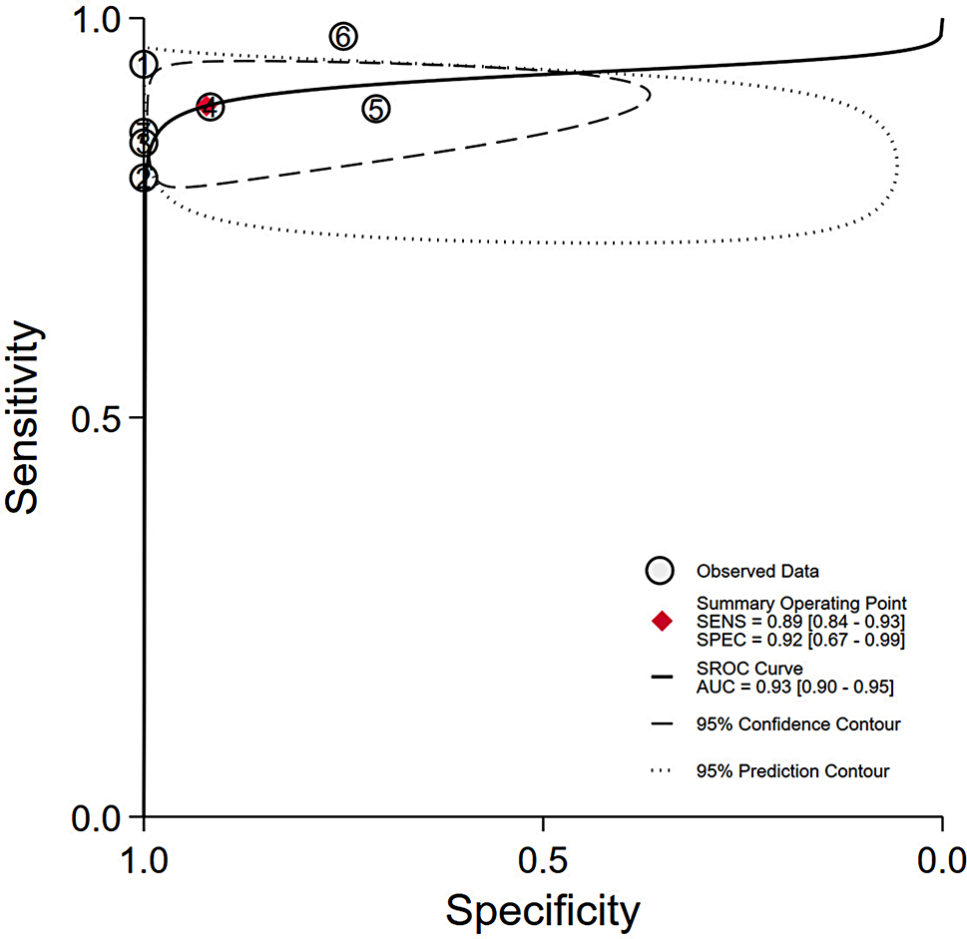
Summary receiver operating characteristic (SROC) and area under the curve (AUC) after combining

**Fig. 6:**
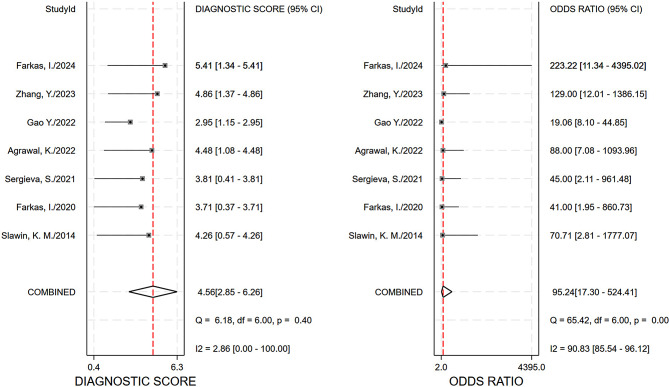
Forest plot for diagnostic odds ratio and score after combination

**Fig. 7:**
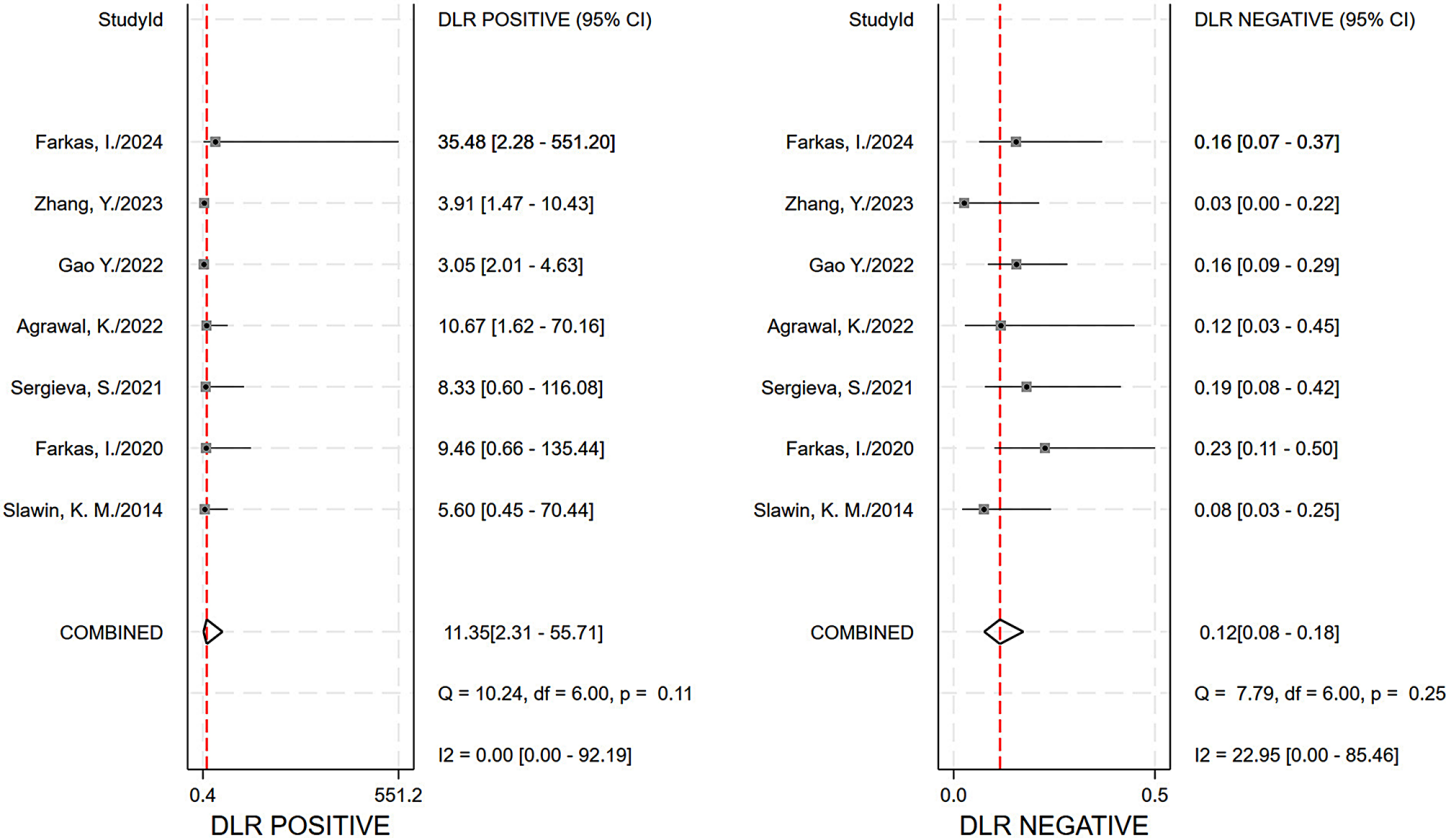
Forest plot of likelihood ratio by combination (LR+, LR-)

**Fig. 8:**
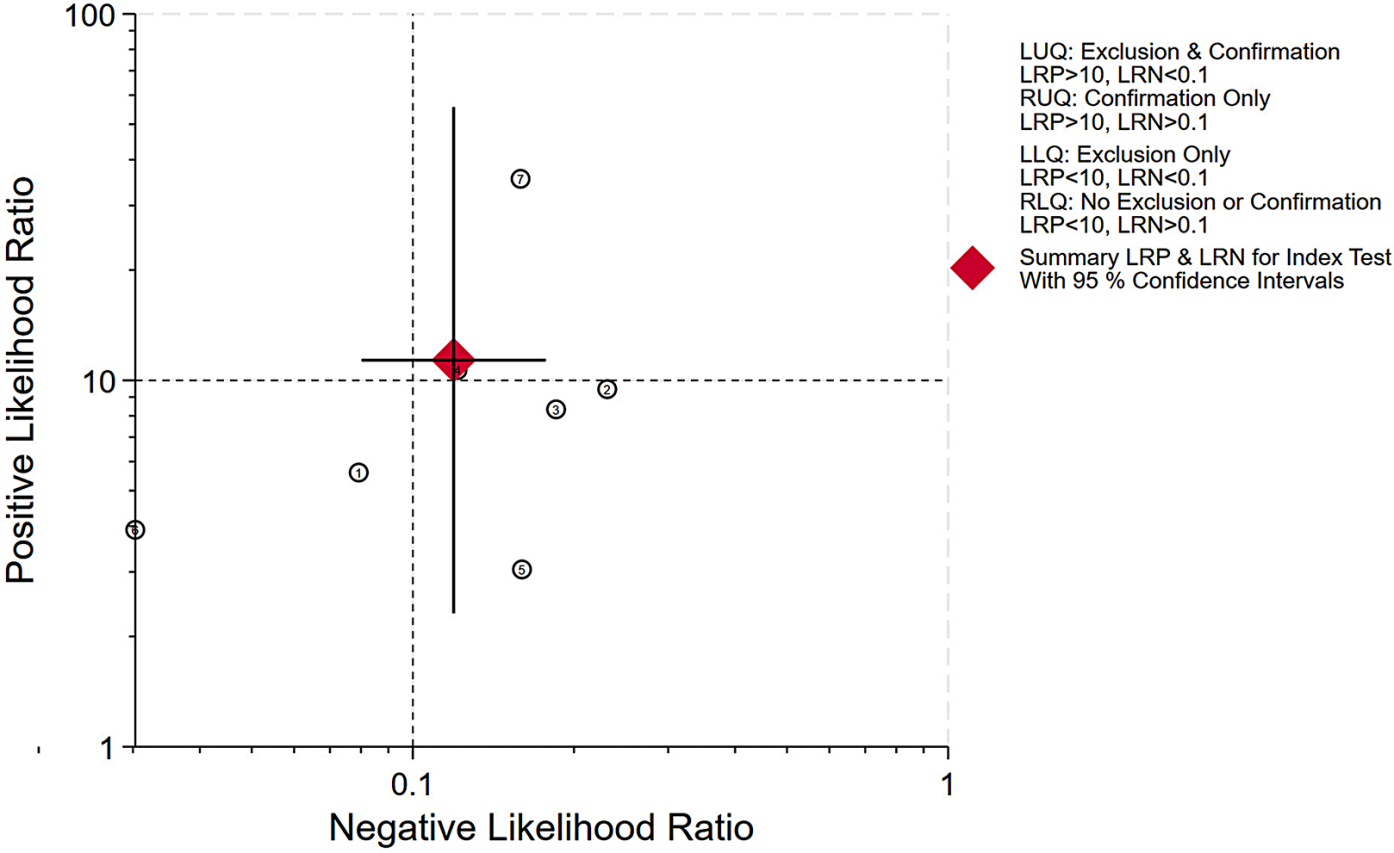
Distribution scatter diagram of the likelihood ratio (LR+/LR-) of each study and combined estimated value. (Top-Right Quadrant: Indicates studies with both high LR + and high LR-, suggesting good diagnostic accuracy)

The pooled sensitivity, specificity and AUC were 0.89 (95% CI, 0.84–0.93), 0.92 (95% CI, 0.67–0.99), and 0.93 (95% CI, 0.90–0.95), respectively. The comprehensive diagnostic odds ratio, diagnostic score, positive likelihood ratio, and negative likelihood ratio were 95.24 (95% CI, 17.30-524.41), 4.56 (95% CI, 2.85–6.26), 11.35 (95% CI, 2.31–55.71), and 0.12 (95% CI, 0.08–0.18) respectively. (Fig. 4– 7). The scatter plot of the likelihood ratios indicated that the combined accuracy of [^99m^Tc]Tc-PSMA SPECT/CT for the diagnosis of prostate cancer was good, with pooled estimates with 95% confidence intervals in the top right quadrant (Fig. 8).

#### Fagan nomogram analysis

**Fig. 9:**
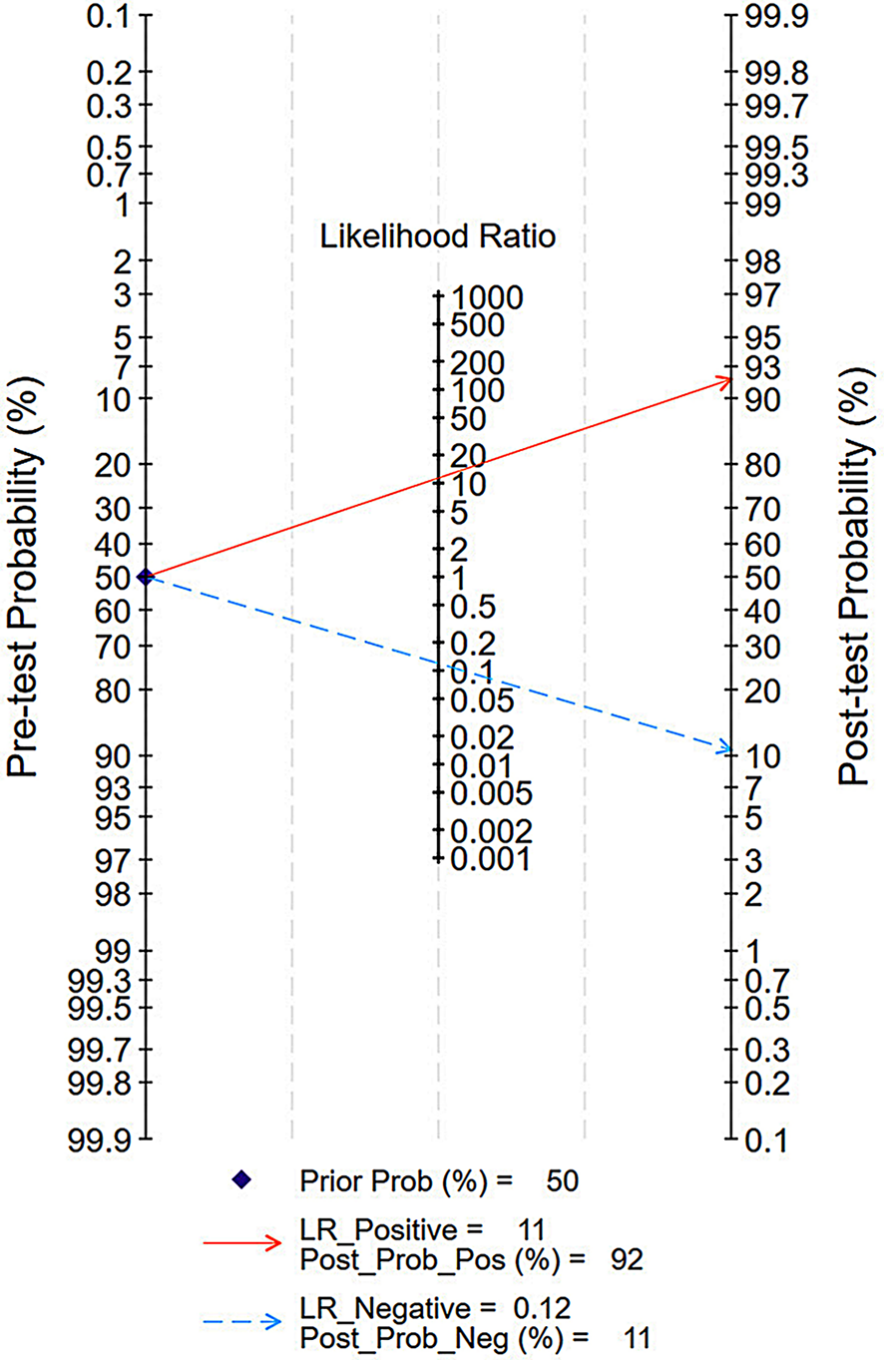
Fagan nomogram of the accuracy of [^99m^Tc]Tc-PSMA SPECT/CT in the diagnosis of prostate cancer

A predicted probability of 50% was used to simulate a clinical situation. This resulted the positive likelihood ratio was 11 and the positive post-test probability was 92%, while the negative likelihood ratio was 0.12 and the negative post-test probability was 11% (Fig. 9).

#### Publication bias analysis

**Fig. 10:**
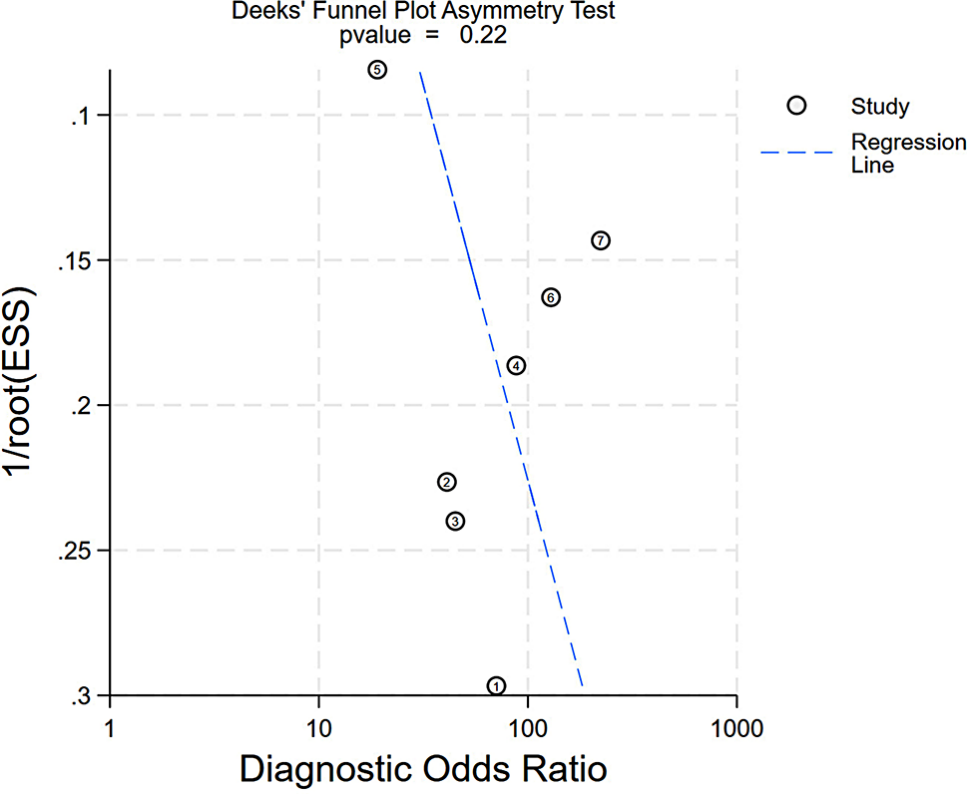
Deeks’ funnel plot of included studies

The Deeks’ funnel plot showed a slope coefficient of 0.22, indicating that there was no relevant publication bias in the included studies (Fig. 10).

## Discussion

Although it has its limitations, SPECT remains the most widely used imaging modality in the world. This is due to the fact that SPECT/CT imaging equipment requires less investment and can be easily extended to remote areas [29, 30]. As radiopharmaceuticals relevant to diagnostic and therapeutic targets are developed in conjunction with acquisition systems, SPECT will continue to play an important role in nuclear medicine imaging [31].

As far as we are aware, no meta-analysis has been published on the diagnostic effectiveness of [^99m^Tc]Tc-PSMA-SPECT/CT for prostate cancer. In our research, six tracers were included in the seven studies, one each of which used [^99m^Tc]Tc-MIP-1404, [^99m^Tc]Tc-PSMA-T4, and [^99m^Tc]Tc-PSMA-11, [^99m^Tc]Tc-PSMA-I&S, and [^99m^Tc]Tc-mas3-ynal-k(Sub-KuE), respectively, as tracers, and two of which used [^99m^Tc]Tc-HYNIC-PSMA, as tracers. All studies showed excellent sensitivity and specificity, and [^99m^Tc]Tc-MIP-1404 showed the best accuracy among all radiotracers (Table 1). [^99m^Tc]Tc-PSMA SPECT/CT showed high sensitivity, specificity, and AUC in the diagnosis of prostate cancer (Figs. 4 and 5). It also performed well in terms of positive likelihood ratio, negative likelihood ratio, and diagnostic score (Figs. 6and 7). Based on these results, we believe that [^99m^Tc]Tc-PSMA SPECT/CT could be considered one of the primary imaging modalities for the diagnosis of prostate cancer.

For the diagnostic work-up of PCa, the European Association of Urology (EAU) guidelines recommend the use of multiparametric magnetic resonance imaging (mpMRI) as standard imaging for biopsy-naïve patients [32]. Performing mpMRI before the initial prostate biopsy may triage patients and reduce unnecessary interventions. MP-MRI can also aid in reducing the overdiagnosis of prostate cancer in patients with subtle clinical symptoms and in increasing the detection rate of prostate cancer in patients with obvious clinical symptoms [33]. However, mpMRI has certain limitations, mpMRI may miss clinically significant intraprostatic tumor lesions, and the occurrence of these limitations include the possibility of false positives or false negatives, as well as the possibility of underestimating tumor volume, which may affect treatment decisions. [34, 35] Relative contraindications to mpMRI such as incompatible implants, which may lead to certain derived limitations in its application compared to CT. [36] Berger et al. [37] compared [^68^Ga]Ga-PSMA-11 PET/CT with mpMRI in localized regional PCa staging, PSMA PET/CT had a higher rate of lesion detection, as well as a higher sensitivity for index lesion localization. A systematic review and meta-analysis conducted by Zhen L. [38] et al. included 29 studies and 8503 patients to assess the accuracy of multiparametric magnetic resonance imaging in diagnosing prostate cancer. The results showed a pooled sensitivity of 0.87 [95% CI, 0.81–0.91] and a pooled specificity of 0.68 [95% CI, 0.56–0.79]. The positive likelihood ratios and negative likelihood ratios were 2.73 [95%CI, 1.90–3.90] and 0.19 [95%CI, 0.14–0.27], respectively. Although direct comparisons with previous meta-analyses are less rigorous, our current meta-analysis suggests that [^99m^Tc]Tc-PSMA SPECT/CT has better diagnostic performance than mpMRI, with better sensitivity, specificity, positive likelihood ratios, and negative likelihood ratios.

PET and SPECT imaging are widely used in clinical diagnostics and therapy [39, 40]. PET is a quantitative imaging tool that appears to surpass SPECT technology, but we cannot simply think which is the better one. PET has the advantage of higher image resolution, fewer attenuation and scattering artifacts, higher sensitivity, and more robust and flexible tracers. SPECT has the advantage of the longer half-life of single photon emitters and their greater ability to target biologically active molecules under certain conditions. However, the high-cost burden limits the availability of PET imaging. In contrast, SPECT imaging is less costly and more readily available in the clinic [31, 41]. A meta-analysis of [^18^F]F-PSMA-1007 PET/CT, [^18^F]F-FDG PET/CT, and [^68^Ga]Ga-PSMA PET/CT for the diagnostic efficacy of prostate cancer by Yu W. et al. [42] showed that the sensitivity and specificity of [^68^Ga]Ga-PSMA PET/CT for the diagnosis of prostate cancer were 0.916 (95%CI, 0.896–0.934) and 0.734 (95%CI, 0.685–0.779), respectively. The positive likelihood ratio was 3.593 (95%CI, 2.986–4.323), the negative likelihood ratio was 0.110 (95%CI, 0.083–0.144) and the AUC was 0.96 (95%CI, 0.92–0.98). Our current analysis indicates that the diagnostic performance of [^99m^Tc]Tc-PSMA SPECT/CT in prostate cancer patients is close to that of [^68^Ga]Ga-PSMA PET/CT. In particular, [^99m^Tc]Tc-PSMA SPECT/CT showed a higher specificity and positive likelihood ratio than [^68^Ga]Ga-PSMA PET/CT, while its sensitivity, AUC, and negative likelihood ratio were relatively low. At the same time, [^99m^Tc]Tc-PSMA SPECT/CT was superior to [^18^F]F-FDG PET/CT in terms of sensitivity, the sensitivity of [^18^F]F-FDG PET/CT is 0.748 (95%CI, 0.698–0.795). [^99m^Tc]Tc-PSMA SPECT/CT was also superior to [^18^F]F-PSMA-1007 PET/CT and [^18^F]F-FDG PET/CT in terms of specificity, the specificities of [^18^F]F-PSMA-1007 PET/CT and [^18^F]F-FDG PET/CT were 0.878 (95%CI, 0.844–0.907) and 0.639 (95%CI, 0.589–0.687), respectively. Although some of literature have shown that [^99m^Tc]Tc-PSMA SPECT/CT for prostate cancer bone metastasis and lymph node metastasis has good prospects, we were unable to study these aspects in this analysis due to the limited number of literature and extractable data that can be collected [13, 18, 43–45].

Three articles were ultimately excluded from the meta-analysis. The article by Duncan, I. et al. [46] was excluded from the study because it highlighted the use of a novel SPECT/CT reconstruction algorithm, which would have led to significant heterogeneity if included in the meta-analysis, and was ultimately excluded from research. Kuru, M. et al. [47] were also excluded from the meta-analysis because they stated that [^99m^Tc]Tc-MIBI-SPECT/CT could not be used as a diagnostic method for prostate cancer. The article by Gatsev, O. et al. [48] was excluded from the study because it did not specify which radiopharmaceuticals were used.

In this meta-analysis, no significant heterogeneity was observed among the included studies. One of the main limitations of our meta-analysis is the small number of studies included, as there are currently only a few clinical trials using [^99m^Tc]Tc-PSMA SPECT/CT for the diagnosis of prostate cancer and our meta-analysis results were based only on patient studies. Subgroup analyses were not performed due to the small amount of literature included. Second, only 2 included studies made explicit statements about blinding. Another limitation is that only 3 studies had sample sizes greater than 50, which may affect the generalizability of the results. Therefore, it is necessary to expand the scope of research in the future, include more literature, and conduct meta-analysis based on lesions to obtain more comprehensive and accurate results. Moreover, this study still needs multi-center, large sample, prospective research to enhance the demonstration intensity.

## Conclusions

This study shows that [^99m^Tc]Tc-PSMA SPECT/CT can indeed supplement clinically useful information, particularly in situations where [^68^Ga]Ga-PSMA PET/CT is not widely available, especially in remote areas. The study findings suggest that [^99m^Tc]Tc-PSMA SPECT/CT has good diagnostic performance for prostate cancer, making it a valuable tool in regions and clinical settings where access to [^68^Ga]Ga-PSMA PET/CT may be limited. In such cases, [^99m^Tc]Tc-PSMA SPECT/CT can help provide important diagnostic information for prostate cancer patients, aiding in their management and treatment decisions.

### Electronic supplementary material

Below is the link to the electronic supplementary material.

Supplementary Material 1

## Data Availability

Full datasets generated during and/or analyzed during the current study are available from the corresponding author on reasonable request. Additional data are available in the supplementary materials.

## Abbreviations

PCa: Prostate cancer
PSMA: Prostate-specific membrane antigen
SPECT: Single photon emission computed tomography
CT: Computed tomography
^68^Ga: Gallium-68
^99m^Tc: Technetium-99 m
PET: Positron emission tomography
DOR: Diagnostic odds ratio
AUC: Area under the curve
SROC: Summary receiver-operating characteristic
LR-: Negative likelihood ratio
LR+: Positive likelihood ratio
^68^Ge/^68^Ga: Germanium-68/gallium-68
^99^Mo/^99m^Tc: Molybdenum/technetiun-99 m
TP: True-positive
FP: False-positive
FN: False-negative
TN: True-negative
QUADAS: Quality assessment of diagnostic accuracy studies
EAU: European association of urology
EU: European unions
US: United states of america
mpMRI: Multiparametric magnetic resonance imaging
