# Supplementary material for: Diagnostic efficacy of [99mTc]Tc-PSMA SPECT/CT for prostate cancer: a meta-analysis
Source: BMC Cancer. 2024 Aug 8;24:982. doi: 10.1186/s12885-024-12734-4 (PMC11312272; doi:10.1186/s12885-024-12734-4)
Supplement: Supplementary file 1 — Supplementary Material 1 [file 12885_2024_12734_MOESM1_ESM.docx]

**Diagnostic Efficacy of [^99m^Tc]Tc-PSMA SPECT/CT for Prostate Cancer:**

**a Meta-Analysis**

**Supplementary materials**

**Search strategy**

| **Database** | **Search strategy** (Search strategy copied from databases) **Search deadline:** 12 July , 2024 |
| --- | --- |
| **Pubmed** | ("Prostatic Neoplasms"[MeSH Terms] OR ("prostate neoplasms"[Title/Abstract] OR "neoplasms prostate"[Title/Abstract] OR "neoplasm prostate"[Title/Abstract] OR "prostate neoplasm"[Title/Abstract] OR "neoplasms prostatic"[Title/Abstract] OR "neoplasm prostatic"[Title/Abstract] OR "prostatic neoplasm"[Title/Abstract] OR "prostate cancer"[Title/Abstract] OR "cancer prostate"[Title/Abstract] OR "cancers prostate"[Title/Abstract] OR "prostate cancers"[Title/Abstract] OR "cancer of the prostate"[Title/Abstract] OR "prostatic cancer"[Title/Abstract] OR "cancer prostatic"[Title/Abstract] OR "cancers prostatic"[Title/Abstract] OR "prostatic cancers"[Title/Abstract] OR "cancer of prostate"[Title/Abstract] OR "prostate carcinoma"[Title/Abstract] OR "prostate carcinomas"[Title/Abstract] OR (("prostat"[All Fields] OR "Prostate"[MeSH Terms] OR "Prostate"[All Fields] OR "prostates"[All Fields] OR "Prostatic"[All Fields] OR "prostatism"[MeSH Terms] OR "prostatism"[All Fields] OR "prostatitis"[MeSH Terms] OR "prostatitis"[All Fields]) AND "Carcinomatosis"[Title/Abstract]) OR (("prostat"[All Fields] OR "Prostate"[MeSH Terms] OR "Prostate"[All Fields] OR "prostates"[All Fields] OR "Prostatic"[All Fields] OR "prostatism"[MeSH Terms] OR "prostatism"[All Fields] OR "prostatitis"[MeSH Terms] OR "prostatitis"[All Fields]) AND "Carcinomatoses"[Title/Abstract]) OR (("prostat"[All Fields] OR "Prostate"[MeSH Terms] OR "Prostate"[All Fields] OR "prostates"[All Fields] OR "Prostatic"[All Fields] OR "prostatism"[MeSH Terms] OR "prostatism"[All Fields] OR "prostatitis"[MeSH Terms] OR "prostatitis"[All Fields]) AND "Epithelioma"[Title/Abstract]) OR (("prostat"[All Fields] OR "Prostate"[MeSH Terms] OR "Prostate"[All Fields] OR "prostates"[All Fields] OR "Prostatic"[All Fields] OR "prostatism"[MeSH Terms] OR "prostatism"[All Fields] OR "prostatitis"[MeSH Terms] OR "prostatitis"[All Fields]) AND "Epitheliomas"[Title/Abstract]) OR "carcinoma of prostate"[Title/Abstract] OR (("Carcinoma"[MeSH Terms] OR "Carcinoma"[All Fields] OR "Carcinomas"[All Fields] OR "carcinoma s"[All Fields]) AND "of prostate"[Title/Abstract]) OR (("Carcinoma"[MeSH Terms] OR "Carcinoma"[All Fields] OR "Epitheliomas"[All Fields] OR "neoplasms, glandular and epithelial"[MeSH Terms] OR ("Neoplasms"[All Fields] AND "glandular"[All Fields] AND "epithelial"[All Fields]) OR "glandular and epithelial neoplasms"[All Fields] OR "Epithelioma"[All Fields]) AND "of prostate"[Title/Abstract]) OR (("Carcinoma"[MeSH Terms] OR "Carcinoma"[All Fields] OR "Epitheliomas"[All Fields] OR "neoplasms, glandular and epithelial"[MeSH Terms] OR ("Neoplasms"[All Fields] AND "glandular"[All Fields] AND "epithelial"[All Fields]) OR "glandular and epithelial neoplasms"[All Fields] OR "Epithelioma"[All Fields]) AND "of prostate"[Title/Abstract]))) AND ("Technetium"[MeSH Terms] OR ("technetium 99m"[Title/Abstract] OR "99m technetium"[Title/Abstract] OR "99mTc"[Title/Abstract] OR "99m-Tc"[Title/Abstract] OR ("99m"[All Fields] AND "Tc"[Title/Abstract]) OR "99m-Tc"[Title/Abstract] OR "99m-Tc"[Title/Abstract] OR "Tc99m"[Title/Abstract] OR "Tc-99m"[Title/Abstract] OR (("cryosphere"[Journal] OR "Tc"[All Fields]) AND "99m"[Title/Abstract]) OR "Tc-99m"[Title/Abstract] OR "Tc-99m"[Title/Abstract])) AND ("tomography, emission computed, single photon"[MeSH Terms] OR ("ct scan single photon emission"[Title/Abstract] OR "ct scan single photon emission"[Title/Abstract] OR (("radioisotopes"[MeSH Terms] OR "radioisotopes"[All Fields] OR "radionuclide"[All Fields] OR "radionuclides"[All Fields] OR "radionuclid"[All Fields] OR "radionuclide s"[All Fields] OR "radionuclidic"[All Fields] OR "radionuclidically"[All Fields] OR "radionuclids"[All Fields]) AND "tomography single photon emission computed"[Title/Abstract]) OR (("radioisotopes"[MeSH Terms] OR "radioisotopes"[All Fields] OR "radionuclide"[All Fields] OR "radionuclides"[All Fields] OR "radionuclid"[All Fields] OR "radionuclide s"[All Fields] OR "radionuclidic"[All Fields] OR "radionuclidically"[All Fields] OR "radionuclids"[All Fields]) AND "tomography single photon emission computed"[Title/Abstract]) OR "tomography single photon emission computed"[Title/Abstract] OR "single photon emission computerized tomography"[Title/Abstract] OR "single photon emission computerized tomography"[Title/Abstract] OR "single photon emission ct scan"[Title/Abstract] OR "single photon emission ct scan"[Title/Abstract] OR "single photon emission computed tomography"[Title/Abstract] OR "emission computed tomography single photon"[Title/Abstract] OR "single photon emission computed tomography"[Title/Abstract] OR "tomography single photon emission computed"[Title/Abstract] OR "SPECT"[Title/Abstract] OR (("tomography, x ray computed"[MeSH Terms] OR ("Tomography"[All Fields] AND "x ray"[All Fields] AND "Computed"[All Fields]) OR "x-ray computed tomography"[All Fields] OR ("cat"[All Fields] AND "Scan"[All Fields]) OR "cat scan"[All Fields]) AND "single photon emission"[Title/Abstract]) OR (("tomography, x ray computed"[MeSH Terms] OR ("Tomography"[All Fields] AND "x ray"[All Fields] AND "Computed"[All Fields]) OR "x-ray computed tomography"[All Fields] OR ("cat"[All Fields] AND "Scan"[All Fields]) OR "cat scan"[All Fields]) AND "single photon emission"[Title/Abstract]) OR "single photon emission computer assisted tomography"[Title/Abstract] OR "single photon emission computer assisted tomography"[Title/Abstract])) AND ("sensitiv*"[Title/Abstract] OR "sensitivity and specificity"[MeSH Terms] OR ("predictive"[Title/Abstract] AND "value*"[Title/Abstract]) OR "predictive value of tests"[MeSH Terms] OR "accuracy*"[Title/Abstract] OR ("Diagnosis"[MeSH Terms] OR ("Diagnoses"[Title/Abstract] OR "Diagnose"[Title/Abstract] OR ("Diagnoses"[Title/Abstract] AND "Examinations"[Title/Abstract]) OR ("Diagnoses"[Title/Abstract] AND "Examination"[Title/Abstract]) OR ("Examination"[Title/Abstract] AND "Diagnoses"[Title/Abstract]) OR ("Examinations"[Title/Abstract] AND "Diagnoses"[Title/Abstract]) OR "antemortem diagnosis"[Title/Abstract] OR "antemortem diagnoses"[Title/Abstract] OR "diagnoses antemortem"[Title/Abstract] OR "diagnosis antemortem"[Title/Abstract] OR "postmortem diagnosis"[Title/Abstract] OR "diagnoses postmortem"[Title/Abstract] OR "diagnosis postmortem"[Title/Abstract] OR "postmortem diagnoses"[Title/Abstract])) OR ("Neoplasm Staging"[MeSH Terms] OR ("cancer staging"[Title/Abstract] OR "staging cancer"[Title/Abstract] OR "staging neoplasm"[Title/Abstract] OR "tumor staging"[Title/Abstract] OR "staging tumor"[Title/Abstract] OR "tnm staging"[Title/Abstract] OR "staging tnm"[Title/Abstract] OR "tnm classification"[Title/Abstract] OR "classifications tnm"[Title/Abstract] OR "classification tnm"[Title/Abstract] OR "tnm classifications"[Title/Abstract] OR "tnm staging system"[Title/Abstract] OR "staging systems tnm"[Title/Abstract] OR "staging system tnm"[Title/Abstract] OR "systems tnm staging"[Title/Abstract] OR "system tnm staging"[Title/Abstract] OR "tnm staging systems"[Title/Abstract]))) Results:228 |
| **Cochrane** | #1 Prostatic Neoplasms Results:9101  #2 (Prostate Neoplasms ):ab,ti,kw OR (Neoplasms, Prostate):ab,ti,kw OR (Neoplasm, Prostate):ab,ti,kw OR (Prostate Neoplasm):ab,ti,kw OR (Neoplasms, Prostatic):ab,ti,kw OR (Neoplasm, Prostatic):ab,ti,kw OR (Prostatic Neoplasm):ab,ti,kw OR (Prostate Cancer):ab,ti,kw OR (Cancer, Prostate):ab,ti,kw OR (Cancers, Prostate):ab,ti,kw OR (Prostate Cancers):ab,ti,kw OR (Cancer of the Prostate):ab,ti,kw OR (Prostatic Cancer):ab,ti,kw OR (Cancer, Prostatic):ab,ti,kw OR (Cancers, Prostatic):ab,ti,kw OR (Prostatic Cancers):ab,ti,kw OR (Cancer of Prostate):ab,ti,kw OR (Prostate Carcinoma):ab,ti,kw OR (Prostate Carcinomas):ab,ti,kw OR (Prostate Carcinomatosis):ab,ti,kw OR (Prostate Carcinomatoses):ab,ti,kw OR (Prostate Epithelioma):ab,ti,kw OR (Prostate Epitheliomas):ab,ti,kw OR (Carcinoma of Prostate ):ab,ti,kw OR (Carcinomas of Prostate ):ab,ti,kw OR (Epitheliomas of Prostate ):ab,ti,kw OR (Epitheliomas of Prostate ):ab,ti,kw 18893  #3 #1 or #2 Results:18907  #4 Technetium Results:1891  #5 (Technetium 99m):ab,ti,kw OR (99m, Technetium ):ab,ti,kw OR (99mTc):ab,ti,kw OR (99m-Tc):ab,ti,kw OR ((99m)Tc):ab,ti,kw OR (99m,Tc):ab,ti,kw OR (99m Tc):ab,ti,kw OR (Tc99m):ab,ti,kw OR (Tc-99m):ab,ti,kw OR (Tc(99m)):ab,ti,kw OR (Tc,99m):ab,ti,kw OR (Tc 99m):ab,ti,kw Results:2735  #6 #4 or #5 Results:2977  #7 Tomography, Emission-Computed, Single-Photon Results:2094  #8 (CT Scan, Single-Photon Emission):ab,ti,kw OR (CT Scan, Single Photon Emission):ab,ti,kw OR (Radionuclide Tomography, Single-Photon Emission-Computed):ab,ti,kw OR (Radionuclide Tomography, Single Photon Emission Computed):ab,ti,kw OR (Tomography, Single-Photon, Emission-Computed):ab,ti,kw OR (Single-Photon Emission Computerized Tomography):ab,ti,kw OR (Single Photon Emission Computerized Tomography):ab,ti,kw OR (Single-Photon Emission CT Scan):ab,ti,kw OR (Single Photon Emission CT Scan):ab,ti,kw OR (Single-Photon Emission-Computed Tomography):ab,ti,kw OR (Emission-Computed Tomography, Single-Photon):ab,ti,kw OR (Single Photon Emission Computed Tomography):ab,ti,kw OR (Tomography, Single-Photon Emission-Computed):ab,ti,kw OR (SPECT):ab,ti,kw OR (CAT Scan, Single-Photon Emission):ab,ti,kw OR (CAT Scan, Single Photon Emission):ab,ti,kw OR (Single-Photon Emission Computer-Assisted Tomography):ab,ti,kw OR (Single Photon Emission Computer Assisted Tomography):ab,ti,kw Results:2745  #9 #7 or #8 Results:2775  #10 (Sensitiv):ab,ti,kw OR (sensitivity and specificity):ab,ti,kw OR (predictive):ab,ti,kw OR (predictive value of tests):ab,ti,kw OR (accuracy):ab,ti,kw Results:71758  #11 Diagnosis Results:214801  #12 (Diagnoses):ab,ti,kw OR (Diagnose):ab,ti,kw OR (Diagnoses and Examinations):ab,ti,kw OR (Diagnoses and Examination):ab,ti,kw OR (Examination and Diagnoses):ab,ti,kw OR (Examinations and Diagnoses):ab,ti,kw OR (Antemortem Diagnosis):ab,ti,kw OR (Antemortem Diagnoses):ab,ti,kw OR (Diagnoses, Antemortem):ab,ti,kw OR (Diagnosis, Antemortem):ab,ti,kw OR (Postmortem Diagnosis):ab,ti,kw OR (Diagnoses, Postmortem):ab,ti,kw OR (Diagnosis, Postmortem):ab,ti,kw OR (Postmortem Diagnoses):ab,ti,kw Results:209992  #13 #11 or #12 Results:216492  #14 Neoplasm Staging Results:11449  #15 (Cancer Staging):ab,ti,kw OR (Staging, Cancer):ab,ti,kw OR (Staging, Neoplasm):ab,ti,kw OR (Tumor Staging):ab,ti,kw OR (Staging, Tumor):ab,ti,kw OR (TNM Staging):ab,ti,kw OR (Staging, TNM):ab,ti,kw OR (TNM Classification):ab,ti,kw OR (Classifications, TNM):ab,ti,kw OR (Classification, TNM):ab,ti,kw OR (TNM Classifications):ab,ti,kw OR (TNM Staging System):ab,ti,kw OR (Staging Systems, TNM):ab,ti,kw OR (Staging System, TNM):ab,ti,kw OR (Systems, TNM Staging):ab,ti,kw OR (System, TNM Staging):ab,ti,kw OR (TNM Staging Systems):ab,ti,kw Results:25154  #16 #14 or #15 Results:25316  #17 #10 or #13 or #16 Results:281085  #18 #3 and #6 and #9 and #17 Results: 6 |
| **EMBASE** | #18. #3 AND #6 AND #9 AND #17 Results: 420 12 Jul 2024  #17. #10 OR #13 OR #16 Results: 8,258,920 12 Jul 2024  #16. #14 OR #15 Results: 483,065 12 Jul 2024  #15. 'neoplasm staging':ab,ti OR 'staging, Results: 20,312 12 Jul 2024  cancer':ab,ti OR 'staging, neoplasm':ab,ti OR  'tumor staging':ab,ti OR 'staging, tumor':ab,ti  OR 'tnm staging':ab,ti OR 'staging, tnm':ab,ti OR  'tnm classification':ab,ti OR 'classifications,  tnm':ab,ti OR 'classification, tnm':ab,ti OR 'tnm  classifications':ab,ti OR 'tnm staging  system':ab,ti OR 'staging systems, tnm':ab,ti OR  'staging system, tnm':ab,ti OR 'systems, tnm  staging':ab,ti OR 'system, tnm staging':ab,ti OR  'tnm staging systems':ab,ti  #14. 'cancer staging' Results: 478,608 12 Jul 2024  #13. #11 OR #12 Results: 7,071,586 12 Jul 2024  #12. 'diagnoses':ab,ti OR 'diagnose':ab,ti OR  'diagnoses and examinations':ab,ti OR 'diagnoses  and examination':ab,ti OR 'examination and  diagnoses':ab,ti OR 'examinations and  diagnoses':ab,ti OR 'antemortem diagnosis':ab,ti  OR 'antemortem diagnoses':ab,ti OR 'diagnoses,  antemortem':ab,ti OR 'diagnosis,  antemortem':ab,ti OR 'postmortem diagnosis':ab,ti  OR 'diagnoses, postmortem':ab,ti OR 'diagnosis,  postmortem':ab,ti OR 'postmortem diagnoses':ab,ti Results: 416,416 12 Jul 2024  #11. 'diagnosis' Results: 6,959,196 12 Jul 2024  #10. 'sensitiv':ab,ti OR 'sensitivity and  specificity':ab,ti OR 'predictive':ab,ti OR  'predictive value of tests':ab,ti OR  'accuracy':ab,ti Results: 1,425,538 12 Jul 2024  #9. #7 OR #8 Results: 92,641 12 Jul 2024  #8. 'ct scan, single-photon emission':ab,ti OR 'ct  scan, single photon emission':ab,ti OR  'radionuclide tomography, single-photon  emission-computed':ab,ti OR 'radionuclide  tomography, single photon emission  computed':ab,ti OR 'tomography, single-photon,  emission-computed':ab,ti OR 'single-photon  emission computerized tomography':ab,ti OR  'single photon emission computerized  tomography':ab,ti OR 'single-photon emission ct  scan':ab,ti OR 'single photon emission ct  scan':ab,ti OR 'single-photon emission-computed  tomography':ab,ti OR 'emission-computed  tomography, single-photon':ab,ti OR 'single  photon emission computed tomography':ab,ti OR  'tomography, single-photon  emission-computed':ab,ti OR 'spect':ab,ti OR 'cat  scan, single-photon emission':ab,ti OR 'cat scan,  single photon emission':ab,ti OR 'single-photon  emission computer-assisted tomography':ab,ti OR  'single photon emission computer assisted  tomography':ab,ti Results: 61,966 12 Jul 2024  #7. 'single photon emission computed tomography' Results: 87,084 12 Jul 2024  #6. #4 OR #5 Results: 81,952 12 Jul 2024  #5. 'technetium':ab,ti OR '99m, technetium':ab,ti OR Results: 68,283 12 Jul 2024  '99mtc':ab,ti OR '99m-tc':ab,ti OR  '(99m)tc':ab,ti OR '99m,tc':ab,ti OR '99m  tc':ab,ti OR 'tc99m':ab,ti OR 'tc-99m':ab,ti OR  'tc(99m)':ab,ti OR 'tc,99m':ab,ti OR 'tc  99m':ab,ti  #4. 'technetium 99m' Results: 44,335 12 Jul 2024  #3. #1 OR #2 Results: 261,601 12 Jul 2024  #2. 'prostate neoplasms':ab,ti OR 'neoplasms,  prostate':ab,ti OR 'neoplasm, prostate':ab,ti OR  'prostate neoplasm':ab,ti OR 'neoplasms,  prostatic':ab,ti OR 'neoplasm, prostatic':ab,ti  OR 'prostatic neoplasm':ab,ti OR 'prostate  cancer':ab,ti OR 'cancer, prostate':ab,ti OR  'cancers, prostate':ab,ti OR 'prostate  cancers':ab,ti OR 'cancer of the prostate':ab,ti  OR 'prostatic cancer':ab,ti OR 'cancer,  prostatic':ab,ti OR 'cancers, prostatic':ab,ti OR  'prostatic cancers':ab,ti OR 'cancer of  prostate':ab,ti OR 'prostate carcinoma':ab,ti OR  'prostate carcinomas':ab,ti OR 'prostate  carcinomatosis':ab,ti OR 'prostate  carcinomatoses':ab,ti OR 'prostate  epithelioma':ab,ti OR 'prostate  epitheliomas':ab,ti OR 'carcinoma of  prostate':ab,ti OR 'carcinomas of prostate':ab,ti  OR 'epitheliomas of prostate':ab,ti Results: 244,501 12 Jul 2024  #1. prostatic AND ('neoplasms'/exp OR neoplasms) Results: 53,680 12 Jul 2024 |
| **Scopus** | ( TITLE-ABS-KEY ( "Prostatic Neoplasms" OR "Prostate Neoplasms " OR "Neoplasms, Prostate" OR "Neoplasm, Prostate" OR "Prostate Neoplasm" OR "Neoplasms, Prostatic" OR "Neoplasm, Prostatic" OR "Prostatic Neoplasm" OR "Prostate Cancer" OR "Cancer, Prostate" OR "Cancers, Prostate" OR "Prostate Cancers" OR "Cancer of the Prostate" OR "Prostatic Cancer" OR "Cancer, Prostatic" OR "Cancers, Prostatic" OR "Prostatic Cancers" OR "Cancer of Prostate" OR "Prostate Carcinoma" OR "Prostate Carcinomas" OR "Prostate Carcinomatosis" OR "Prostate Carcinomatoses" OR "Prostate Epithelioma" OR "Prostate Epitheliomas" OR "Carcinoma of Prostate " OR "Carcinomas of Prostate " OR "Epitheliomas of Prostate " OR "Epitheliomas of Prostate" ) AND TITLE-ABS-KEY ( "Technetium" OR "Technetium 99m" OR "99m, Technetium " OR "99mTc" OR "99m-Tc" OR "(99m)Tc" OR "99m,Tc" OR "99m Tc" OR "Tc99m" OR "Tc-99m" OR "Tc(99m)" OR "Tc,99m" OR "Tc 99m" ) AND TITLE-ABS-KEY ( "Tomography, Emission-Computed, Single-Photon" OR "CT Scan, Single-Photon Emission" OR "CT Scan, Single Photon Emission" OR "Radionuclide Tomography, Single-Photon Emission-Computed" OR "Radionuclide Tomography, Single Photon Emission Computed" OR "Tomography, Single-Photon, Emission-Computed" OR "Single-Photon Emission Computerized Tomography" OR "Single Photon Emission Computerized Tomography" OR "Single-Photon Emission CT Scan" OR "Single Photon Emission CT Scan" OR "Single-Photon Emission-Computed Tomography" OR "Emission-Computed Tomography, Single-Photon" OR "Single Photon Emission Computed Tomography" OR "Tomography, Single-Photon Emission-Computed" OR "SPECT" OR "CAT Scan, Single-Photon Emission" OR "CAT Scan, Single Photon Emission" OR "Single-Photon Emission Computer-Assisted Tomography" OR "Single Photon Emission Computer Assisted Tomography" ) AND TITLE-ABS-KEY ( "Sensitiv" OR "sensitivity and specificity" OR "predictive" OR "predictive value of tests" OR "accuracy" OR "Diagnosis" OR "Diagnoses" OR "Diagnose" OR "Diagnoses and Examinations" OR "Diagnoses and Examination" OR "Examination and Diagnoses" OR "Examinations and Diagnoses" OR "Antemortem Diagnosis" OR "Antemortem Diagnoses" OR "Diagnoses, Antemortem" OR "Diagnosis, Antemortem" OR "Postmortem Diagnosis" OR "Diagnoses, Postmortem" OR "Diagnosis, Postmortem" OR "Postmortem Diagnoses" OR "Neoplasm Staging" OR "Cancer Staging" OR "Staging, Cancer" OR "Staging, Neoplasm" OR "Tumor Staging" OR "Staging, Tumor" OR "TNM Staging" OR "Staging, TNM" OR "TNM Classification" OR "Classifications, TNM" OR "Classification, TNM" OR "TNM Classifications" OR "TNM Staging System" OR "Staging Systems, TNM" OR "Staging System, TNM" OR "Systems, TNM Staging" OR "System, TNM Staging" OR "TNM Staging Systems" ) ) Results: 354 |
| **Ovid** | 1 (Prostatic Neoplasms or Prostate Neoplasms or Neoplasms, Prostate or Neoplasm, Prostate or Prostate Neoplasm or Neoplasms, Prostatic or Neoplasm, Prostatic or Prostatic Neoplasm or Prostate Cancer or Cancer, Prostate or Cancers, Prostate or Prostate Cancers or Cancer of the Prostate or Prostatic Cancer or Cancer, Prostatic or Cancers, Prostatic or Prostatic Cancers or Cancer of Prostate or Prostate Carcinoma or Prostate Carcinomas or Prostate Carcinomatosis or Prostate Carcinomatoses or Prostate Epithelioma or Prostate Epitheliomas or Carcinoma of Prostate or Carcinomas of Prostate or Epitheliomas of Prostate or Epitheliomas of Prostate).ti,ab,kw. Results:165696  2 (Technetium or Technetium 99m or 99m, Technetium or 99mTc or 99m-Tc or 99m,Tc or 99m Tc or Tc99m or Tc-99m or Tc,99m or Tc 99m).ti,ab,kw. Results:46514  3 (Tomography, Emission-Computed, Single-Photon or CT Scan, Single-Photon Emission or CT Scan, Single Photon Emission or Radionuclide Tomography, Single-Photon Emission-Computed or Radionuclide Tomography, Single Photon Emission Computed or Tomography, Single-Photon, Emission-Computed or Single-Photon Emission Computerized Tomography or Single Photon Emission Computerized Tomography or Single-Photon Emission CT Scan or Single Photon Emission CT Scan or Single-Photon Emission-Computed Tomography or Emission-Computed Tomography, Single-Photon or Single Photon Emission Computed Tomography or Tomography, Single-Photon Emission-Computed or SPECT or CAT Scan, Single-Photon Emission or CAT Scan, Single Photon Emission or Single-Photon Emission Computer-Assisted Tomography or Single Photon Emission Computer Assisted Tomography).ti,ab,kw. Results:38435  4 (((Sensitiv or sensitivity) and specificity) or predictive or predictive value of tests or accuracy).ti,ab,kw. Results:1158081  5 (((((((((Diagnosis or Diagnoses or Diagnose or Diagnoses) and Examinations) or Diagnoses) and Examination) or Examination) and Diagnoses) or Examinations) and Diagnoses) or Antemortem Diagnosis or Antemortem Diagnoses or Diagnoses, Antemortem or Diagnosis, Antemortem or Postmortem Diagnosis or Diagnoses, Postmortem or Diagnosis, Postmortem or Postmortem Diagnoses).ti,ab,kw. 19971  6 (Neoplasm Staging or Cancer Staging or Staging, Cancer or Staging, Neoplasm or Tumor Staging or Staging, Tumor or TNM Staging or Staging, TNM or TNM Classification or Classifications, TNM or Classification, TNM or TNM Classifications or TNM Staging System or Staging Systems, TNM or Staging System, TNM or Systems, TNM Staging or System, TNM Staging or TNM Staging Systems).ti,ab,kw. Results: 18433  7 4 or 5 or 6 Results: 1189298  8 1 and 2 and 3 and 7 Results: 64 |
| **Web of Science** | 1: Prostatic Neoplasms (Topic) OR Prostate Neoplasms (Topic) OR Neoplasms, Prostate (Topic) OR Neoplasm, Prostate (Topic) OR Prostate Neoplasm (Topic) OR Neoplasms, Prostatic (Topic) OR Neoplasm, Prostatic (Topic) OR Prostatic Neoplasm (Topic) OR Prostate Cancer (Topic) OR Cancer, Prostate (Topic) OR Cancers, Prostate (Topic) OR Prostate Cancers (Topic) OR Cancer of the Prostate (Topic) OR Prostatic Cancer (Topic) OR Cancer, Prostatic (Topic) OR Cancers, Prostatic (Topic) OR Prostatic Cancers (Topic) OR Cancer of Prostate (Topic) OR Prostate Carcinoma (Topic) OR Prostate Carcinomas (Topic) OR Prostate Carcinomatosis (Topic) OR Prostate Carcinomatoses (Topic) OR Prostate Epithelioma (Topic) OR Prostate Epitheliomas (Topic) OR Carcinoma of Prostate (Topic) OR Carcinomas of Prostate (Topic) OR Epitheliomas of Prostate (Topic) OR Epitheliomas of Prostate (Topic) and Preprint Citation Index (Exclude – Database)  Results: 437998  2: Technetium (Topic) OR Technetium 99m (Topic) OR 99m, Technetium (Topic) OR 99mTc (Topic) OR 99m-Tc (Topic) OR (99m)Tc (Topic) OR 99m,Tc (Topic) OR 99m Tc (Topic) OR Tc99m (Topic) OR Tc-99m (Topic) OR Tc(99m) (Topic) OR Tc,99m (Topic) OR Tc 99m (Topic) and Preprint Citation Index (Exclude – Database) Results: 99492  3: Tomography, Emission-Computed, Single-Photon (Topic) OR CT Scan, Single-Photon Emission (Topic) OR CT Scan, Single Photon Emission (Topic) OR Radionuclide Tomography, Single-Photon Emission-Computed (Topic) OR Radionuclide Tomography, Single Photon Emission Computed (Topic) OR Tomography, Single-Photon, Emission-Computed (Topic) OR Single-Photon Emission Computerized Tomography (Topic) OR Single Photon Emission Computerized Tomography (Topic) OR Single-Photon Emission CT Scan (Topic) OR Single Photon Emission CT Scan (Topic) OR Single-Photon Emission-Computed Tomography (Topic) OR Emission-Computed Tomography, Single-Photon (Topic) OR Single Photon Emission Computed Tomography (Topic) OR Tomography, Single-Photon Emission-Computed (Topic) OR SPECT (Topic) OR CAT Scan, Single-Photon Emission (Topic) OR CAT Scan, Single Photon Emission (Topic) OR Single-Photon Emission Computer-Assisted Tomography (Topic) OR Single Photon Emission Computer Assisted Tomography (Topic) and Preprint Citation Index (Exclude – Database)  Results: 88760  4: Sensitiv (Topic) OR sensitivity and specificity (Topic) OR predictive (Topic) OR predictive value of tests (Topic) OR accuracy (Topic) and Preprint Citation Index (Exclude – Database)  Results: 3201369  5: Diagnosis (Topic) OR Diagnoses (Topic) OR Diagnose (Topic) OR Diagnoses and Examinations (Topic) OR Diagnoses and Examination (Topic) OR Examination and Diagnoses (Topic) OR Examinations and Diagnoses (Topic) OR Antemortem Diagnosis (Topic) OR Antemortem Diagnoses (Topic) OR Diagnoses, Antemortem (Topic) OR Diagnosis, Antemortem (Topic) OR Postmortem Diagnosis (Topic) OR Diagnoses, Postmortem (Topic) OR Diagnosis, Postmortem (Topic) OR Postmortem Diagnoses (Topic) and Preprint Citation Index (Exclude – Database)  Results: 7073080  6: Neoplasm Staging (Topic) OR Cancer Staging (Topic) OR Staging, Cancer (Topic) OR Staging, Neoplasm (Topic) OR Tumor Staging (Topic) OR Staging, Tumor (Topic) OR TNM Staging (Topic) OR Staging, TNM (Topic) OR TNM Classification (Topic) OR Classifications, TNM (Topic) OR Classification, TNM (Topic) OR TNM Classifications (Topic) OR TNM Staging System (Topic) OR Staging Systems, TNM (Topic) OR Staging System, TNM (Topic) OR Systems, TNM Staging (Topic) OR System, TNM Staging (Topic) OR TNM Staging Systems (Topic) and Preprint Citation Index (Exclude – Database) Results: 805993  7: #4 OR #5 OR #6 and Preprint Citation Index (Exclude – Database)  Results: 9951389  8: #1 AND #2 AND #3 AND #7 and Preprint Citation Index (Exclude – Database)  Results: 395 |
